# The use of common salt for the treatment of pyogenic granuloma

**DOI:** 10.1016/j.jdcr.2024.08.016

**Published:** 2024-09-01

**Authors:** Ghadah Alhammad, Maha Albaraka, Hend Alotaibi, Abdulaziz Madani

**Affiliations:** a Department of Dermatology, College of Medicine, King Saud University, Riyadh, Saudi Arabia; b College of Medicine, King Saud University, Riyadh, Saudi Arabia

**Keywords:** pyogenic granuloma, salt

## Introduction

Pyogenic granuloma (PG) is an acquired benign capillary proliferation of the skin and mucosa,^1^ although the term is a misnomer as the lesion is neither infectious in etiology nor granulomatous in histology. PG may occur at any age but is more common among children and young adults. Both males and females are affected, although females are affected more frequently, potentially due to the vascular effects of estrogen and progesterone.^2^ We report the complete resolution of a PG lesion and alleviation of associated symptoms in a 53-year-old female patient following the home application of topical salt.

## Case report

**Fig 1:**
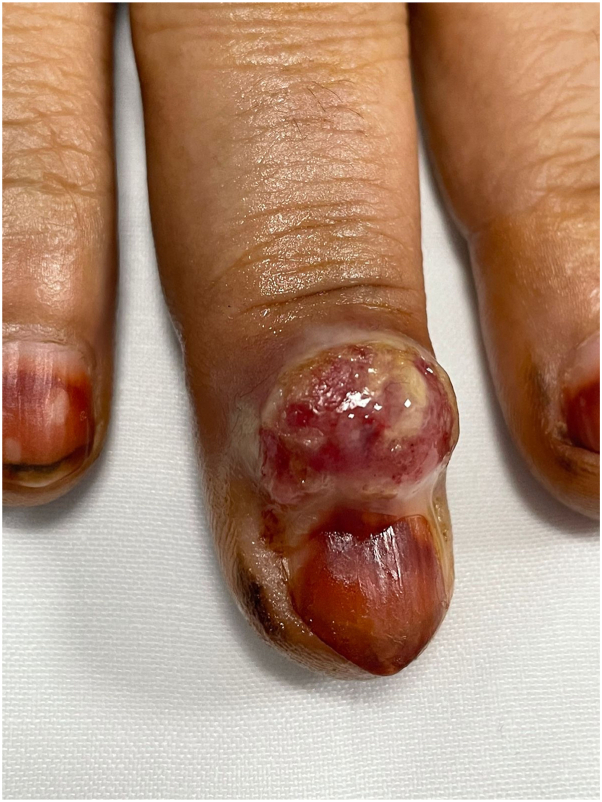
Solitary bright erythematous dome-shaped nodule with superimposed yellowish crusting over the dorsal periungual area of the right middle finger. Henna stain over the fingernails is shown.

A 53-year-old female patient presented to the emergency department with a rapidly growing tumor on the right middle finger with recurrent episodes of bleeding. She was known to have diabetes mellitus, hypertension, dyslipidemia, and hypothyroidism. The patient reported that swelling had started after self-induced trauma around 20 days prior to her presentation. The patient denied having any recent changes in her medications, and she had no history of similar conditions. Examination revealed a solitary bright erythematous dome-shaped nodule with yellowish crusting superimposed over the dorsal periungual area of the right middle finger (Fig 1).

**Fig 2:**
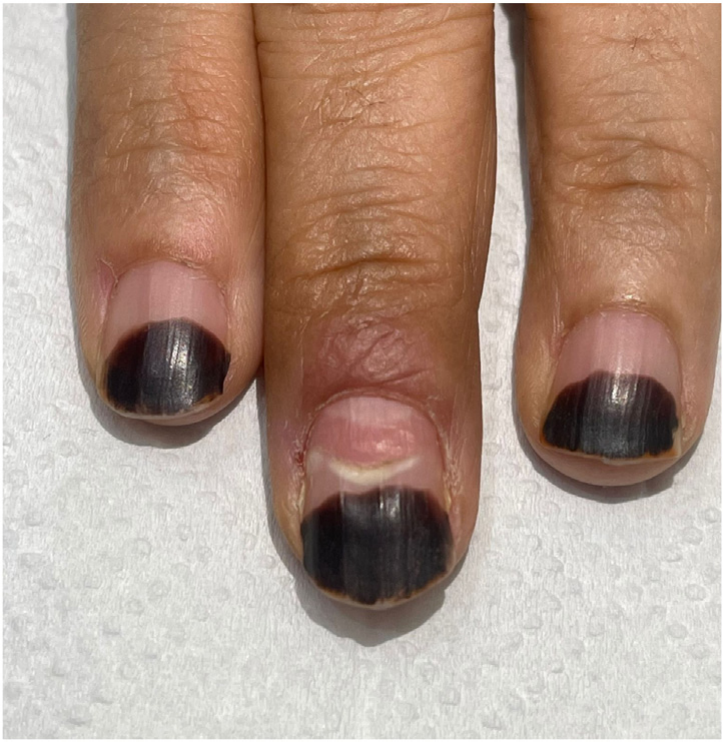
Complete resolution of the lesion within 3 weeks following daily application of common salt.

Based on the history and clinical appearance of the lesion, a diagnosis of PG was made. Various treatment options were discussed with the patient, and she was offered treatment with sodium chloride (common salt). This treatment involved covering the entire lesion with salt after protecting the perilesional skin with white soft paraffin, followed by application of a simple dressing. The patient was instructed to repeat the steps daily at home. After 3 weeks of daily salt application, the lesion resolved completely (Fig 2), and pain and movement restriction of the finger also resolved. The patient was satisfied with the result and reported no side effects.

## Discussion

The etiology of lobular capillary hemangioma, which is often referred to as PG, remains uncertain. Evidence has demonstrated an association between PG and variant factors, including pre-existing trauma or irritation, hormonal effects, and medications such as oral retinoids.^1^ The classic presentation of PG is a rapidly growing, solitary, friable, red papule that frequently ulcerates and bleeds with minor trauma.

PG is diagnosed clinically; however, histological confirmation is necessary if the diagnosis is not certain or in older patients with large lesion sizes to rule out the possibility of PG-like amelanotic melanoma.^3^ Nonetheless, given the rapid presentation in our patient and the rarity of melanoma in our region, biopsy has not been obtained. PG has a distinctive histology of lobular aggregates of capillary-sized vessels with central feeding vessels.^2^^,^^4^

Various treatments are available for the management of PG, including noninvasive options like laser surgery, electrodesiccation, curettage, liquid nitrogen cryotherapy, sclerotherapy, topical silver nitrate, and topical imiquimod, as well as invasive approaches such as surgical excision and shave excision.^2^^,^^4^ However, these treatments are frequently associated with recurrence due to inadequate excision or eradication of the lesions. Salt therapy has emerged as a novel treatment for PG and has been validated in a prospective open-label uncontrolled study by Daruwalla et al.^4^ They enrolled 50 patients with PG, and a small amount of salt was applied to the lesions after the application of soft white paraffin around the margins of the lesion to prevent the irritation of normal skin. The lesions were then covered with adhesive surgical tape.

Remarkably, the lesions resolved within 14.77 days (range 6-38 days). In all cases, the lesions fully resolved without leaving any scars, and 94% of patients reported an immediate reduction in the bleeding tendency of the lesions. Only one patient reported recurrence after 11 months of initial resolution. Similar case reports yielded similar outcomes and results that were consistent with those of Daruwalla et al.^5^, ^6^, ^7^ Our patient followed the same instructions mentioned by Daruwalla et al and exhibited complete resolution along with healthy growth of her nail after 3 weeks of daily application, thus supporting the results in the literature.

The proposed mechanism of salt therapy suggests that it creates a hyperosmolar environment within the affected area, which causes the granuloma to desiccate and eventually shrink.^2^^,^^4^^,^^8^ While PGs can develop at any age, they are notably more common in children, adolescents, and pregnant women. The average age of onset in children is approximately 6.7 years. PG constitutes approximately 0.5% of all skin nodules in childhood and has a predilection for appearance on the face.^2^ In this population, salt therapy may be beneficial due to minimal scarring (particularly on the face) and a low recurrence rate. These benefits might also be in other patient populations, such as those with concerns about scarring and elderly individuals who might not tolerate other procedures.

In conclusion, salt therapy is a cost-effective and widely accessible therapeutic option for PG that has minimal adverse effects. The feasibility of salt application allows for self-administration by patients at home. In addition, it has the capacity to resolve PG lesions rapidly with lower reported recurrence rates than other options reported in the literature. This makes it a more preferrable option for specific populations such as pediatrics, the elderly, and those who have concerns about scarring. Nevertheless, further comprehensive studies are necessary to validate its effectiveness on a larger scale.

## Conflicts of interest

None disclosed.
